# Preterm Delivery and Postpartum Substance Use: Implications for Maternal Mental Health in Obstetric Care

**DOI:** 10.7759/cureus.100263

**Published:** 2025-12-28

**Authors:** Freida P Raj, Eduardo Espiridion, Xuezhi (Daniel) Jiang

**Affiliations:** 1 Obstetrics and Gynecology, Drexel College of Medicine, Philadelphia, USA; 2 Psychiatry, Reading Hospital, Tower Health, West Reading, USA; 3 Obstetrics and Gynecology, Reading Hospital, Tower Health, West Reading, USA

**Keywords:** alcohol, cannabis, maternal screening, maternal substance use, nicotine, opioid, postpartum depression, preterm delivery, preterm labor and obstetric care

## Abstract

Introduction

Preterm delivery is an obstetric complication linked to an increased risk of postpartum depression (PPD), which often goes undiagnosed. Individuals with PPD may self-medicate to manage depressive symptoms. While postpartum substance use is well-documented, few studies have assessed whether preterm delivery is associated with new-onset substance use. A greater understanding of birth trauma as a factor for new-onset substance use in postpartum women can broaden our understanding of postpartum mood disorders and propel greater inquiry into mental health care in peri- and postpartum women.

Methods

A retrospective cohort study was conducted using TriNetX to assess whether preterm delivery increases the risk of new-onset postpartum substance use. Propensity score matching was performed based on age, age at delivery, and race. Patients with a history of substance use disorder were excluded. Two postpartum periods (1-180 and 181-365 days) were analyzed using risk and hazard ratios (HRs), Kaplan-Meier analysis, and log-rank tests. The proportional hazards assumption was tested using Schoenfeld’s residuals.

Results

After propensity score matching, 104,187 participants were included per cohort. Preterm delivery was associated with an increased risk of new-onset substance use across all four substances. In the early postpartum period, HRs for preterm vs. full-term delivery were: nicotine 2.193 (95% CI: 1.888, 2.547), cannabis 2.486 (95% CI: 1.773, 3.484), opioids 4.040 (95% CI: 2.245, 7.273), and alcohol 2.451 (95% CI: 1.547, 3.883). These associations persisted, though slightly attenuated, in the late postpartum period.

Discussion

Preterm delivery was linked to increased new-onset postpartum substance use across substances and time periods, with the strength of association varying by substance and timing. These identified associations between preterm labor and postpartum substance use may suggest a link between preterm birth and coping behaviors such as self-medication. Proactive postpartum screening and trauma-informed care are crucial for improving outcomes in patients with preterm delivery. Maternal care must integrate trauma-informed care and behavioral health services.

## Introduction

Preterm labor is defined as uterine contraction and cervical change occurring prior to 37 weeks of gestation [1]. Preterm labor with preterm delivery is a birthing trauma defined as the delivery of an infant before 37 weeks of gestation [1]. Preterm delivery often introduces sudden medical interventions, neonatal complications, and heightened maternal stress, making it a significant event that can impact a mother’s psychological well-being both immediately postpartum and long after [2]. A population study of over 80,000 women in the US found a strong association between preterm delivery and maternal depressive symptoms and hopelessness [3]. Postpartum depression (PPD) is typically defined as the onset of depressive symptoms occurring between the first and 12th months following childbirth [4]. PPD remains both vastly understudied and significantly underdiagnosed. Estimates suggest that more than 50% of affected individuals go undiagnosed, often due to the tendency of postpartum patients to ignore, minimize, or deny their symptoms [5]. A retrospective chart review found that among women enrolled in a substance abuse treatment program, 43.7% of those screened positive for PPD [6], highlighting the frequent overlap between mood disorders and substance use.

The frequent co-occurrence of PPD and substance use may be partly explained by the phenomenon of self-medication. Studies have documented perinatal patients using substances such as tobacco and other drugs to relieve symptoms of perinatal depression and anxiety [7]. The substances examined in this study include alcohol, opioids, cannabis, and nicotine. Current literature has explored maternal use of each of these substances in various contexts. A national survey found that new mothers have a high prevalence of alcohol and illicit drug use [8]. The authors suggested that since depression correlates with substance use, mothers with PPD may be at a higher risk of substance use [8]. Supporting this, a separate study followed 300 women who quit nicotine during pregnancy and found that 50% relapsed within one year postpartum, despite their expressed desires to remain abstinent [9]. This highlights how these stressors during childbirth and the postpartum period can drive substance use even among those highly motivated to remain abstinent.

Additional research continues to highlight the prevalence of self-medication in mood disorders. In states where cannabis is legal, postpartum women were found to be 1.83 times more likely to use it [10]. In fact, out of a group of participants who use cannabis to manage health conditions, 70% of them reported using cannabis daily postpartum [10]. A population study reported that in opioid-naive women, there is a 0.67% incidence of persistent opioid use at one year postpartum overall [11].

Another body of research centers on the effects of maternal substance use disorder on neonates. Studies show that infants born to women with substance use disorders are at an increased risk for adverse neonatal outcomes such as preterm birth, low birth weight, and other respiratory, neurologic, and gastrointestinal disorders [12]. Despite well-documented effects of maternal substance use on neonates, few studies have examined the bidirectional nature of substance use, mood disorders, and pregnancy. A greater understanding of birth trauma as a factor for new-onset substance use in postpartum women can broaden our understanding of postpartum mood disorders and propel greater inquiry into mental health care in peri- and postpartum women.

Although the current literature has vast data on prenatal onset of substance use and its effects on the child, less attention has been paid to how specific obstetric factors, such as preterm delivery, may influence the development of new-onset substance use in the postpartum period. This retrospective cohort study aims to evaluate whether preterm delivery is associated with an increased risk of new-onset postpartum substance use by utilizing a large, propensity-matched dataset from TriNetX [13].

## Materials and methods

Study design

A retrospective cohort study was conducted using the TriNetX platform. The inclusion and exclusion criteria were outlined using ICD-10-CM codes. All patients included were 18 years or older. Cohort 1 consisted of patients with a documented encounter for a preterm delivery with preterm labor (ICD-10 code O60.1) and excluded those with full-term delivery or preterm labor without delivery (ICD-10 codes O80, O60.0, O60.2). Cohort 2 consisted of patients with a documented encounter for full-term, uncomplicated delivery (ICD-10 code O80) and excluded those with any diagnosis of preterm labor (ICD-10 codes O60.0-O60.2). Both groups excluded patients with a history of substance use disorder before the date of delivery (ICD-10 codes F10-F19), allowing us to determine newly documented ICD-10 diagnoses. Patients with multiple gestations (ICD-10 code O30) were excluded, as multiple gestation is a known risk factor for preterm delivery and could confound the association under investigation. Substance use outcomes were defined by the presence of clinical encounters indicating the use of alcohol (ICD-10 code F10), opioids (ICD-10 code F11), cannabis (ICD-10 code F12), or nicotine (ICD-10 code F17). These outcomes were assessed across two distinct postpartum timeframes: early postpartum (1-180 days) and late postpartum (181-365 days).

Data source

This study utilized data from TriNetX, a global health research platform that aggregates de-identified electronic medical records from over 110 million patients [13]. The platform was used to aggregate data during June 2025. The dataset analyzed was drawn from 146 healthcare organizations, with records primarily sourced from electronic health records dating back to 2006 [13]. TriNetX provides comprehensive clinical data, including diagnoses, medications, procedures, laboratory values, and patient demographics [13].

Participants

This study included data from 146 healthcare organizations, encompassing over 110 million patient records. The two cohorts were subsequently propensity score-matched based on demographic variables, including age, age at the time of the event, and race. This matching process resulted in 104,187 participants in each cohort, yielding a total sample size of 208,374. The control cohort comprised patients who experienced full-term, uncomplicated deliveries with no documented history of substance use. The comparative cohort consisted of patients with preterm labor and delivery, also without a prior history of substance use.

Statistical methods

The "Compare Cohorts" analysis tool on the TriNetX platform was used to evaluate measures of association and survival between the two cohorts [13]. Statistical analyses included the calculation of risk ratios, hazard ratios (HRs), Kaplan-Meier survival curves, and the log-rank test. The analysis of the two cohorts was conducted over two distinct postpartum time intervals: 1-180 days and 181-365 days after delivery.

## Results

Sample characteristics

The data from each cohort were propensity-matched for age, age at the time of delivery, race, and ethnicity before analysis. Propensity score matching ensures demographic comparability between both cohorts. After propensity matching, each cohort included 104,187 patients. In both cohorts, the mean age at the time of delivery was 29.6 (Table 1).

**Table 1:** Characteristics of patients with full-term and preterm delivery, excluding those with a history of substance use, after matching cohorts on age, age at the time of delivery, race, and ethnicity (n=104,187). Demographic characteristics of patients included in the dataset. 1: cohort with preterm labor and preterm delivery without a history of substance use (n=104,187); 2: cohort with an encounter for a full-term delivery without a history of substance use (n=104,187). Age is presented as mean ± standard deviation, while categorical variables are shown as percentages.

Cohort	Demographics	Mean ± SD	Patients	% of cohort	P-value	Standard difference
1	Current age	37.6 +/- 7.8	104,187	100%	0.756	0.001
2		37.6 +/- 7.9	104,187	100%		
1	Age at index	29.6 +/- 6.5	104,187	100%	0.610	0.002
2		29.6 +/- 6.6	104,187	100%		
1	White		47,837	45.9%	0.878	0.001
2			47,802	45.9%		
1	American Indian or Alaska Native		633	0.6%	0.485	0.003
2			658	0.6%		
1	Unknown race		26,272	25.2%	0.920	<0.001
2			26,292	25.2%		
1	Native Hawaiian or other Pacific Islander		832	0.8%	0.541	0.003
2			857	0.8%		
1	Unknown ethnicity		41,345	39.7%	0.318	0.004
2			41,568	39.9%		
1	Not Hispanic or Latino		44,539	42.7%	0.495	0.003
2			44,385	42.6%		
1	Hispanic or Latino		18,303	17.6%	0.691	0.002
2			18,234	17.5%		
1	Black or African American		16,239	15.6%	0.909	0.001
2			16,220	15.6%		
1	Other Race		5,468	5.2%	0.497	0.003
2			5,399	5.2%		
1	Asian		6,906	6.6%	0.641	0.002
2			6,959	6.7%		

Nicotine 

1-180 Days Postpartum

In the early postpartum period, nicotine use was observed in 529 patients in the preterm cohort (n=104,187) and 254 patients in the full-term cohort (n=104,187), with corresponding risks of 0.005 and 0.002, respectively. The absolute risk difference between the two cohorts was 0.003 (95% CI: 0.002, 0.003; z=9.846; p<0.001), indicating a statistically significant lower risk of nicotine use in the full-term group. The survival probability between the preterm and full-term cohorts was 99.33% and 99.69%, respectively.

The risk ratio was 2.083 (95% CI: 1.794, 2.418), and the odds ratio was 2.088 (95% CI: 1.798, 2.426), which suggests an increased risk and odds of nicotine use in patients who were delivered preterm. Kaplan-Meier survival analysis showed an HR of 2.193 (95% CI: 1.888, 2.547), with a significant log-rank test (χ²=111.390, df=1, p<0.001), which further highlights the statistically significant difference in time to first nicotine use between cohorts (Table 2).

**Table 2:** Measures of association and comparison for nicotine use in the early postpartum period for patients with preterm or full-term delivery. This table presents comparative risk estimates and association measures for nicotine use in the early postpartum period. For each cohort, the table reports the number of patients experiencing the outcome, the absolute risk, and the survival probability. Measures of association include risk difference, risk ratio, odds ratio, and hazard ratio, each reported with corresponding 95% CI. The z-values and p-values reflect the statistical significance of each effect estimate, and chi-square values (with degrees of freedom) correspond to the log-rank test used to compare survival distributions between the cohorts. These data, taken together, quantify the magnitude of the difference in substance use between patients with preterm delivery and full-term delivery.

Cohort	Patients in the cohort	Patients with outcome	Risk	Survival probability	Metric	Value	95% CI	z	p-value	Chi-squared	df
Preterm	104,187	529	0.005	99.33%	Risk difference	0.003	(0.002, 0.003)	9.846	<0.001	-	-
Full-term	104,187	254	0.002	99.69%	Risk ratio	2.083	(1.794, 2.418)	-	-	-	-
					Odds ratio	2.088	(1.798, 2.426)	-	-	-	-
					Hazard ratio	2.193	(1.888, 2.547)	-	0.805	0.061	1
					Rank-log test	-	-	-	<0.001	111.390	1

181-365 Days Postpartum

In the late postpartum time period, 347 patients in the preterm cohort (n=103,659) and 201 in the full-term cohort (n=103,933) had a history of nicotine use, yielding risks of 0.003 and 0.002, respectively. TriNetX excluded 528 patients from the preterm cohort and 254 patients from the full-term cohort because they had new-onset nicotine use before 181 days postpartum. The risk difference was 0.001 (95% CI: 0.001, 0.002; z=6.276; p<0.001), indicating a statistically significant lower risk in the full-term group. The survival probability between the preterm and full-term cohorts was 99.48% and 99.72%, respectively.

Risk and odds ratios were 1.731 (95% CI: 1.455, 2.059) and 1.733 (95% CI: 1.457, 2.063), respectively. Kaplan-Meier analysis demonstrated an HR of 1.889 (95% CI: 1.588, 2.247), with a significant log-rank test (χ²=53.236, df=1, p<0.001), once again demonstrating a meaningful delay in nicotine use among full-term patients (Table 3).

**Table 3:** Measures of association and comparison for nicotine use in the late postpartum period for patients with preterm or full-term delivery. This table presents comparative risk estimates and association measures for nicotine use in the late postpartum period. For each cohort, the table reports the number of patients experiencing the outcome, the absolute risk, and the survival probability. Measures of association include risk difference, risk ratio, odds ratio, and hazard ratio, each reported with corresponding 95% CI. The z-values and p-values reflect the statistical significance of each effect estimate, and chi-square values (with degrees of freedom) correspond to the log-rank test used to compare survival distributions between the cohorts. These data, taken together, quantify the magnitude of difference in substance use between patients with preterm delivery and full-term delivery.

Cohort	Patients in the cohort	Patients with outcome	Risk	Survival Probability	Metric	Value	95% CI	z	p-value	Chi-squared	df
Preterm	103,659	347	0.003	99.48%	Risk Difference	0.001	(0.001, 0.002)	6.276	<0.001	-	-
Full-Term	103,933	201	0.002	99.72%	Risk Ratio	1.731	(1.455, 2.059)	-	-	-	-
					Odds Ratio	1.733	(1.457, 2.063)	-	-	-	-
					Hazard Ratio	1.889	(1.588, 2.247)	-	0.407	0.687	1
					Rank-Log Test	-	-	-	<0.001	53.236	1

Cannabis

1-180 Days Postpartum

In the first 180 days after delivery, cannabis use was reported in 113 patients in the preterm cohort (n=104,187) and 48 patients in the full-term cohort (n=104,187), with corresponding risks of 0.001 and <0.001, respectively. Although the absolute risk was low in both groups, the difference was statistically significant (risk difference=0.001; 95% CI: 0.000, 0.001; z=5.159; p<0.001). The preterm group had a noticeably lower survival probability (99.85%) compared to the full-term group (99.94%).

The risk ratio was 2.354 (95% CI: 1.680, 3.299) and the odds ratio was 2.356 (95% CI: 1.680, 3.302), indicating that patients who delivered preterm were more likely to use cannabis in the early postpartum period. Kaplan-Meier survival analysis showed an HR of 2.486 (95% CI: 1.773, 3.484) with a significant log-rank test (χ²=29.911, df=1, p<0.001), indicating a significantly higher hazard of cannabis use in the preterm group (Table 4).

**Table 4:** Measures of association and comparison for cannabis use in the early postpartum period for patients with preterm or full-term delivery. This table presents comparative risk estimates and association measures for cannabis use in the early postpartum period. For each cohort, the table reports the number of patients experiencing the outcome, the absolute risk, and the survival probability. Measures of association include risk difference, risk ratio, odds ratio, and hazard ratio, each reported with corresponding 95% CI. The z-values and p-values reflect the statistical significance of each effect estimate, and chi-square values (with degrees of freedom) correspond to the log-rank test used to compare survival distributions between the cohorts. These data, taken together, quantify the magnitude of difference in substance use between patients with preterm delivery and full-term delivery.

Cohort	Patients in the cohort	Patients with outcome	Risk	Survival probability	Metric	Value	95% CI	z	p-value	Chi-squared	df
Preterm	104,187	113	0.001	99.85%	Risk difference	0.001	(0.000, 0.001)	5.125	<0.001	-	-
Full-term	104,187	48	<0.001	99.94%	Risk ratio	2.354	(1.680, 3.299)	-	-	-	-
					Odds ratio	2.356	(1.680, 3.302)	-	-	-	-
					Hazard ratio	2.486	(1.773, 3.484)	-	0.863	0.030	1
					Rank-log test	-	-	-	<0.001	29.911	1

181-365 Days Postpartum

For the late postpartum period, cannabis use was reported in 62 patients in the preterm cohort (n=104,075) and 30 in the full-term cohort (n=104,139). TriNetX excluded 112 patients from the preterm cohort and 48 patients from the full-term cohort because they had new-onset cannabis use before 181 days postpartum. Although the absolute differences were minimal, the risk of cannabis use remained statistically lower in the full-term group. The preterm group had a marginally lower survival probability (99.91%) compared to the full-term group (99.96%).

The risk ratio was 2.068 (95% CI: 1.337, 3.198) and the odds ratio was 2.069 (95% CI: 1.338, 3.199), which suggests a higher risk and odds of cannabis use in patients who delivered preterm. Kaplan-Meier survival analysis showed an HR of 2.255 (95% CI: 1.458, 3.487), with a significant log-rank test (χ²=14.121, df=1, p<0.001), which, though minimal, still indicates a higher hazard of cannabis use in the preterm group (Table 5). 

**Table 5:** Measures of association and comparison for cannabis use in the late postpartum period for patients with preterm or full-term delivery. This table presents comparative risk estimates and association measures for cannabis use in the late postpartum period. For each cohort, the table reports the number of patients experiencing the outcome, the absolute risk, and the survival probability. Measures of association include risk difference, risk ratio, odds ratio, and hazard ratio, each reported with corresponding 95% CI. The z-values and p-values reflect the statistical significance of each effect estimate, and chi-square values (with degrees of freedom) correspond to the log-rank test used to compare survival distributions between the cohorts. These data, taken together, quantify the magnitude of difference in substance use between patients with preterm delivery and full-term delivery.

Cohort	Patients in the cohort	Patients with outcome	Risk	Survival probability	Metric	Value	95% CI	z	p-value	Chi-squared	df
Preterm	104,075	62	0.001	99.91%	Risk difference	<0.001	(0.000, 0.000)	3.340	0.001	-	-
Full-term	104,139	30	0.000	99.96%	Risk ratio	2.068	(1.337, 3.198)	-	-	-	-
					Odds ratio	2.069	(1.338, 3.199)	-	-	-	-
					Hazard ratio	2.255	(1.458, 3.487)	-	0.458	0.551	1
					Rank-log test	-	-	-	<0.001	14.121	1

Opioid

1-180 Days Postpartum

In the early postpartum period, opioid use was indicated in 54 patients in the preterm cohort (n=104,187) and 14 patients in the full-term cohort (n=104,187). Though the absolute difference in incidence was small, the risk difference was statistically significant at <0.001 (95% CI: 0.000 to 0.001; z=4.852, p<0.001). The survival probability was slightly lower in the preterm cohort (99.93%) compared to the full-term cohort (99.98%).

The risk ratio was 3.857 (95% CI: 2.143, 6.943) and the odds ratio was 3.859 (95% CI: 2.143, 6.946), indicating that preterm patients were more likely to use opioids compared to their full-term counterparts. Kaplan-Meier survival analysis revealed an HR of 4.040 (95% CI: 2.245, 7.273), with a significant log-rank test result (χ²=25.431, df=1, p<0.001), suggesting a significantly higher hazard of opioid use in the preterm cohort (Table 6).

**Table 6:** Measures of association and comparison for opioid use in the early postpartum period for patients with preterm or full-term delivery. This table presents comparative risk estimates and association measures for opioid use in the early postpartum period. For each cohort, the table reports the number of patients experiencing the outcome, the absolute risk, and the survival probability. Measures of association include risk difference, risk ratio, odds ratio, and hazard ratio, each reported with corresponding 95% CI. The z-values and p-values reflect the statistical significance of each effect estimate, and chi-square values (with degrees of freedom) correspond to the log-rank test used to compare survival distributions between the cohorts. These data, taken together, quantify the magnitude of difference in substance use between patients with preterm delivery and full-term delivery.

Cohort	Patients in the cohort	Patients with outcome	Risk	Survival probability	Metric	Value	95% CI	z	p-value	Chi-squared	df
Preterm	104,187	54	0.001	99.93%	Risk difference	<0.001	(0.000, 0.001)	4.852	<0.001	-	-
Full-term	104,187	14	0.000	99.98%	Risk ratio	3.857	(2.143, 6.943)	-	-	-	-
					Odds ratio	3.859	(2.143, 6.946)	-	-	-	-
					Hazard ratio	4.040	(2.245, 7.273)	-	0.192	1.704	1
					Rank-log test	-	-	-	<0.001	25.431	1

181-365 Days Postpartum

In the late postpartum window, 22 individuals in the preterm group (n=104,133) and 10 in the full-term group (n=104,173) had reported opioid use. TriNetX excluded 54 patients from the preterm cohort and 14 patients from the full-term cohort because they had new-onset opioid use prior to 181 days postpartum. The risk difference was again statistically significant (<0.001; 95% CI: 0.000 to 0.000; z=2.123, p=0.034), though the absolute incidence remained low in both groups, and the strength of association was weaker than in the early period. The preterm group had a marginally lower survival probability (99.97%) compared to the full-term group (99.99%). 

The risk ratio was 2.201 (95% CI: 1.042, 4.647) and the odds ratio was 2.201 (95% CI: 1.042, 4.648), indicating borderline statistical significance. Survival analysis yielded an HR of 3.428 (95% CI: 1.464, 8.024), suggesting a continued higher hazard of opioid use in the preterm group. The difference in survival distributions was statistically significant (log-rank χ²=9.130, df=1, p=0.003), though with a less pronounced difference than during the earlier postpartum phase (Table 7).

**Table 7:** Measures of association and comparison for opioid use in the late postpartum period for patients with preterm or full-term delivery. This table presents comparative risk estimates and association measures for opioid use in the late postpartum period. For each cohort, the table reports the number of patients experiencing the outcome, the absolute risk, and the survival probability. Measures of association include risk difference, risk ratio, odds ratio, and hazard ratio, each reported with corresponding 95% CI. The z-values and p-values reflect the statistical significance of each effect estimate, and chi-square values (with degrees of freedom) correspond to the log-rank test used to compare survival distributions between the cohorts. These data, taken together, quantify the magnitude of difference in substance use between patients with preterm delivery and full-term delivery.

Cohort	Patients in the cohort	Patients with outcome	Risk	Survival probability	Metric	Value	95% CI	z	p-value	Chi-squared	df
Preterm	104,133	22	0.000	99.97%	Risk difference	<0.001	(0.000, 0.000)	2.123	0.034	-	-
Full-term	104,173	10	0.000	99.99%	Risk ratio	2.201	(1.042, 4.647)	-	-	-	-
					Odds ratio	2.201	(1.042, 4.648)	-	-	-	-
					Hazard ratio	3.428	(1.464, 8.024)	-	0.169	1.889	1
					Rank-log test	-	-	-	0.003	9.130	1

Alcohol

1-180 Days Postpartum

In the early postpartum period, 60 patients in the preterm cohort (n=104,187) and 26 patients in the full-term cohort (n=104,187) had documented alcohol use. This corresponded to a risk of 0.001 and <0.001 for preterm and full-term patients, respectively. The risk difference, although minimal, was statistically significant at <0.001 (95% CI: 0.000-0.001; z=3.667; p<0.001). The preterm group also had a lower survival probability (99.92%) compared to the full-term group (99.97%).

The risk ratio was 2.308 (95% CI: 1.457, 3.656) and the odds ratio was 2.308 (95% CI: 1.457, 3.658), which suggests an increased risk and odds of alcohol use in patients who delivered preterm. Kaplan-Meier survival analysis showed an HR of 2.451 (95% CI: 1.547, 3.883), with a significant log-rank test (χ²=15.575, df=1, p<0.001), indicating a significantly higher hazard of alcohol use in the preterm group (Table 8).

**Table 8:** Measures of association and comparison for alcohol use in the early postpartum period for patients with preterm or full-term delivery. This table presents comparative risk estimates and association measures for alcohol use in the early postpartum period. For each cohort, the table reports the number of patients experiencing the outcome, the absolute risk, and the survival probability. Measures of association include risk difference, risk ratio, odds ratio, and hazard ratio, each reported with corresponding 95% CI. The z-values and p-values reflect the statistical significance of each effect estimate, and chi-square values (with degrees of freedom) correspond to the log-rank test used to compare survival distributions between the cohorts. These data, taken together, quantify the magnitude of difference in substance use between patients with preterm delivery and full-term delivery.

Cohort	Patients in the cohort	Patients with outcome	Risk	Survival probability	Metric	Value	95% CI	z	p-value	Chi-squared	df
Preterm	104,187	60	0.001	99.92%	Risk difference	<0.001	(0.000, 0.001)	3.667	<0.001	-	-
Full-term	104,187	26	<0.001	99.97%	Risk ratio	2.308	(1.457, 3.656)	-	-	-	-
					Odds ratio	2.308	(1.457, 3.658)	-	-	-	-
					Hazard ratio	2.451	(1.547, 3.883)	-	0.727	0.122	1
					Rank-log test	-	-	-	<0.001	15.575	1

181-365 Days Postpartum

During the late postpartum window, 55 patients in the preterm cohort (n=104,128) and 22 patients in the full-term cohort (n=104,161) had documented alcohol use. TriNetX excluded 59 patients from the preterm cohort and 26 patients from the full-term cohort because they had new-onset alcohol use prior to 181 days postpartum. Although the risk incidence was minimal, the risk difference was statistically significant at <0.001 (95% CI: 0.000-0.000; z=3.763; p<0.001). Additionally, the survival probability for the preterm cohort was less than for the full-term cohort at 99.92% and 99.97%, respectively.

The risk ratio was 2.501 (95% CI: 1.525, 4.100) and the odds ratio was 2.502 (95% CI: 1.526, 4.102), indicating that patients who delivered preterm were more likely to use alcohol in the late postpartum period. Kaplan-Meier survival analysis showed an HR of 2.724 (95% CI: 1.661, 4.465), with a significant log-rank test (χ²=17.140, df=1, p<0.001), indicating a significantly higher hazard of alcohol use in the preterm group (Table 9).

**Table 9:** Measures of association and comparison for alcohol use in the late postpartum period for patients with preterm or full-term delivery. This table presents comparative risk estimates and association measures for alcohol use in the late postpartum period. For each cohort, the table reports the number of patients experiencing the outcome, the absolute risk, and the survival probability. Measures of association include risk difference, risk ratio, odds ratio, and hazard ratio, each reported with corresponding 95% confidence intervals. The z-values and p-values reflect the statistical significance of each effect estimate, and chi-square values (with degrees of freedom) correspond to the log-rank test used to compare survival distributions between the cohorts. These data, taken together, quantify the magnitude of difference in substance use between patients with preterm delivery and full-term delivery.

Cohort	Patients in the cohort	Patients with outcome	Risk	Survival probability	Metric	Value	95% CI	z	p-value	Chi-squared	df
Preterm	104,128	55	0.001	99.92%	Risk difference	<0.001	(0.000, 0.000)	3.763	<0.001	-	-
Full-term	104,161	22	0.000	99.97%	Risk ratio	2.501	(1.525, 4.100)	-	-	-	-
					Odds ratio	2.502	(1.526, 4.102)	-	-	-	-
					Hazard ratio	2.724	(1.661, 4.465)	-	0.678	0.172	1
					Rank-log test	-	-	-	<0.001	17.140	1

## Discussion

Preterm delivery was consistently associated with increased postpartum substance use across different substances and time periods, although the magnitude of association varied slightly by substance and timing. Nicotine use exhibited the largest absolute risk difference, followed by cannabis use. While there was a statistically significantly higher hazard of alcohol or opioid use among preterm patients, the absolute differences were modest, although this may reflect the relatively low incidence of these outcomes rather than a lack of a meaningful difference.

These identified associations between preterm labor and postpartum substance use may suggest a link between preterm birth and coping behaviors such as self-medication. However, the pattern can also be a result of unmeasured risk factors such as socioeconomic status and other social determinants of health. The association observed persisted across both time periods examined (1-180 days and 181-365 days). Preterm birth likely indicates a psychosocial stressor that could increase susceptibility to maladaptive coping strategies, including new-onset substance use. Concerns about neonatal health and unexpected medical interventions may additionally compound the physical and mental stress of early labor. This study aligns with existing literature that demonstrates a link between preterm delivery and PPD. One scoping review found that eight out of 12 studies indicated an increased risk for PPD among mothers with preterm deliveries [14]. Another population study found that even after excluding mothers with mood disorders, mothers with preterm delivery had higher levels of PPD symptoms for up to 12 months after delivery [15]. These authors support the idea that preterm labor is a source of birthing trauma that contributes to the diagnosis of PPD.

The results of this analysis add to the growing body of evidence that proactive, long-term, and robust screening is necessary for new mothers as they navigate the postpartum period, especially those who have undergone birth traumas such as preterm labor. Maternal care must integrate trauma-informed care and behavioral health services. Given the dynamic nature of mental health in the postpartum period, hospitals and clinics should implement proactive screening by flagging patient charts to ensure mental health and substance use histories are reviewed at follow-up visits for at least one year after discharge from their delivery encounter.

It is essential to recognize that both substance use disorders and preterm birth are strongly linked to shared social determinants of health, particularly low socioeconomic status (SES). Since factors such as poverty, lack of access to healthcare, and chronic stress are highly prevalent among both groups, it is difficult to isolate the independent effect of coded substance use. This shared etiology suggests that SES may be acting as a powerful unmeasured confounder. Further research should explore how socioeconomic factors modulate the risk of PPD and substance use. Future studies should also examine the rates of PPD associated with other forms of birth trauma, including perineal injury, pelvic organ damage, postpartum hemorrhage, and other obstetric complications. These findings underscore the need for interventional trials to develop better strategies to address PPD in high-risk populations.

Strengths and limitations

This study utilized a large, diverse sample from the TriNetX electronic health record platform, which aggregates medical data from over 146 global healthcare institutions [13]. The standardization of ICD-10 codes improved the consistency and objectivity of exclusion and inclusion criteria as well as the outcomes observed across institutions. Given the ability to track longitudinal substance use patterns between early and late postpartum, this study was able to provide insight into short- and long-term trajectories of risk.

This study has several limitations. As a retrospective analysis of medical data, it cannot determine causality but rather the strength of association. Using an EHR system makes the data subject to potential data inaccuracies and coding variability. Underdiagnosis and misdiagnosis of key variables, including substance use, preterm delivery, and full-term delivery, may affect the validity of findings. These data cannot fully account for misclassification bias resulting from the transient cessation or intentional concealment of substance use during the prenatal period. Therefore, some cases categorized as "new-onset" in the postpartum phase could represent a resumption of preexisting use, potentially biasing the calculated incidence rate. Another notable limitation is the inability to ascertain the patient's entry date into the EHR system. This constraint prohibits a definitive determination of whether a documented substance use history existed prior to the study period, potentially leading to an overestimation of newly documented ICD-10 substance use codes.

Despite exclusion criteria, unmeasured confounders such as increased exposure to healthcare systems, social determinants of health, and insurance could influence outcomes. Mothers of preterm infants, by necessity, experience more frequent and sustained engagement with the healthcare system. This increased surveillance heightens the likelihood that a preexisting or concurrent substance use issue will be identified and subsequently documented. Another limitation is that although two postpartum intervals were analyzed, these may not fully capture the dynamic nature of maternal mental health and the long-lasting effects of birth trauma.

## Conclusions

This study used TriNetX to create a large retrospective cohort to investigate the co-occurrences of birth traumas and substance use disorder across four different substances. The results indicated that there was a statistically significant variance in new-onset substance use for patients who experienced a preterm birth versus patients who had a full-term delivery. This emphasizes the need for robust screening for PPD and the integration of maternal care with mental health services. By implementing trauma-informed care in screening mothers for PPD, we can address mental health challenges that mothers face in the postpartum period. Maternal mental health is a multifaceted issue, and the healthcare system must work alongside mothers to be proactive instead of reactive.
